# Intensive Care to Facilitate Organ Donation: Insights From the French Guidelines

**DOI:** 10.3389/ti.2025.14840

**Published:** 2025-11-21

**Authors:** Matthieu Le Dorze, Aurore Armand, Julien Charpentier, Lisa Humbertjean, Benjamin Zuber

**Affiliations:** 1 Department of Anesthesiology and Critical Care, Lariboisiere Hospital, FHU PROMICE, DMU Parabol, INSERM UMR942, MASCOT, Hôpital Lariboisière, Université Paris Cité, APHP Nord, Paris, France; 2 Emergency Department, Angers University Hospital, Angers, République des Savoirs- Lettres, Sciences, Philosophie - USR3608- ED540- ENS-PSL, Paris, France; 3 Service de médecine intensive-réanimation, Hôpital Cochin, Assistance Publique - Hôpitaux de Paris, Paris, France; 4 Neurology, CHRU de Nancy, Nancy, France; 5 Medical Intensive Care Unit, Hôpital Foch, Suresnes, France

**Keywords:** intensive care to facilitate organ donation, devastating brain injury, brain death, controlled donation after circulatory death, antemortem intervention

## Abstract

Intensive care to facilitate organ donation (ICOD) is being discussed internationally without reaching a consensus. The aim of this paper is to share with the community the recently published French ICOD guidelines, focusing on two main ethical issues: the ethical acceptability of antemortem interventions during the ICOD process, and the ethical acceptability of considering controlled donation after circulatory death during the ICOD process. These issues raised by the tension between end-of-life care and the possibility of OD deserve to be addressed as they challenge the consideration of ICOD as a routine part of end-of-life care.

## Introduction

Intensive care to facilitate organ donation (ICOD) has been defined in the Spanish guidelines as the initiation or continuation of intensive care in patients with a devastating brain injury, in whom medical and surgical treatments for curative purpose have been deemed futile and who are considered possible organ donors with the aim of incorporating the option of donation after brain death (DBD) into their end-of-life care plan [1, 2]. ICOD supports the concept that donation should considered as a routine at the end of life [3–5]. This strategy has been developed mainly in Spain [1, 2, 6, 7] and is being discussed internationally without reaching a consensus [8–14]. The idea that the intensive care measures are not solely made in the best interests of the patient, but must also consider the interests of a third party, in this case the patient awaiting for transplantation, is challenging [15, 16].

In France, ICOD was mentioned as early as 2010 in expert recommendations on stroke management with a weak agreement [17]. However, no specific French recommendations on this topic have existed until now. The French ICOD guidelines have just been published, taking into account the specific framework and the professional codes (Supplementary file 1) [18]. A working group was set up that included experts in intensive care medicine and organ donation (OD), as well as experts in the humanities and representatives of healthcare system users and of donors’ families. The French guidelines are organized into several sections. The first section addresses the key elements required to implement an ICOD procedure within a healthcare facility. This includes prior institutional reflection on staff information and training; identification by the OD team of relevant partner departments (such as hospital emergency departments, neurology and neurovascular intensive care units); and the collaborative development and formalization of a local protocol with the OD team, partner departments, and ICU staff. A second section defines the ICOD pathway and describes the successive steps involved, which we presented in the first part of this manuscript. All the authors participated in this national-level working groups organized by the *Agence de la Biomédecine* to develop these recommendations.

As each country has its specific legal regulations, medical codes, and cultural backgrounds [19], we would like to share these guidelines with the community by focusing on the two main issues: are antemortem intervention for donation ethically acceptable within the ICOD pathway ? How should controlled donation after the circulatory determination of death (cDCD) be integrated within the ICOD pathway?

## The French ICOD Guidelines

In the French guidelines, ICOD is defined as the process by which a patient with a devastating brain injury and a severe coma (Glasgow Coma Scale score <8 or rapidly worsening coma), for whom no therapeutic plan is available (i.e., whom medical and surgical treatments for curative purpose have been deemed futile [20]), and who has a high likelihood of progressing to brain death (BD), is admitted to intensive care unit (ICU) solely for the purpose of OD. This process relies on a multidisciplinary approach and includes the evaluation of the clinical context, logistical support, and several discussions with the patient’s relatives- including an *early interview* to inform about ICOD and to seek consent and authorization. This process involves the patient, their relatives, and the caregivers in end-of-life support. The *early interview*, as part of an ongoing communication, is defined as a discussion between the caregivers and the relatives with the aim of raising the possibility of post-mortem organ and tissue donation, and documenting any refusal expressed by the patient during his or her lifetime, as well as the relatives’ agreement with this approach. Legally, France applies an opt-out system: everyone is presumed to be a donor unless they have expressed their refusal to be a donor during their lifetime. This refusal can be expressed in three ways: registration on a national refusal registry (which can only be consulted after death has been formally declared), written testimony, or oral testimony. In practice, an interview with the relatives is always conducted. Its purpose is first to gather any possible testimony of refusal expressed by the patient during their lifetime, to comply with the legal framework. Beyond this legal requirement, engaging with relatives is also essential to ensure that the donation process remains acceptable to them, to avoid conflicts, and to preserve trust through full transparency in what is an emotionally very difficult moment. In France, as in many other countries, national guidelines and best practice recommendations explicitly require that these interviews be systematically conducted by the organ donation personnel. This ensures both compliance with the legal framework and alignment with international standards aiming to optimize donation discussions.

The French definition of ICOD differs slightly from the original Spanish definition in several important respects. In Spain, ICOD is understood to include both the initiation and continuation of intensive care for the purpose of OD. In contrast, the French guidelines specifically address the management of patients with devastating brain injury who are not yet in the ICU but rather in partner services (such as emergency departments or neurology units), who, in the vast majority of cases, are not intubated at the time of initial assessment, and for whom a decision has been made not to admit them to the ICU for a therapeutic plan. They do not include the referral of patients who have already been admitted to the ICU after having been placed on invasive ventilation by emergency teams, either outside or inside the hospital, and for whom the ICU team has decided - either at the time of the initial assessment or after several hours or days of treatment - that no therapeutic plan is possible, but that intensive care could nonetheless be initiated solely for the purpose of OD [20]. For these patients already admitted to the ICU, it is recommended to prioritize discussing OD after the BD diagnosis, while informing the family of the imminent (i.e., no early interview). An early mention of OD should only occur if the family specifically inquires about the continuation of intensive care.

**FIGURE 1:**
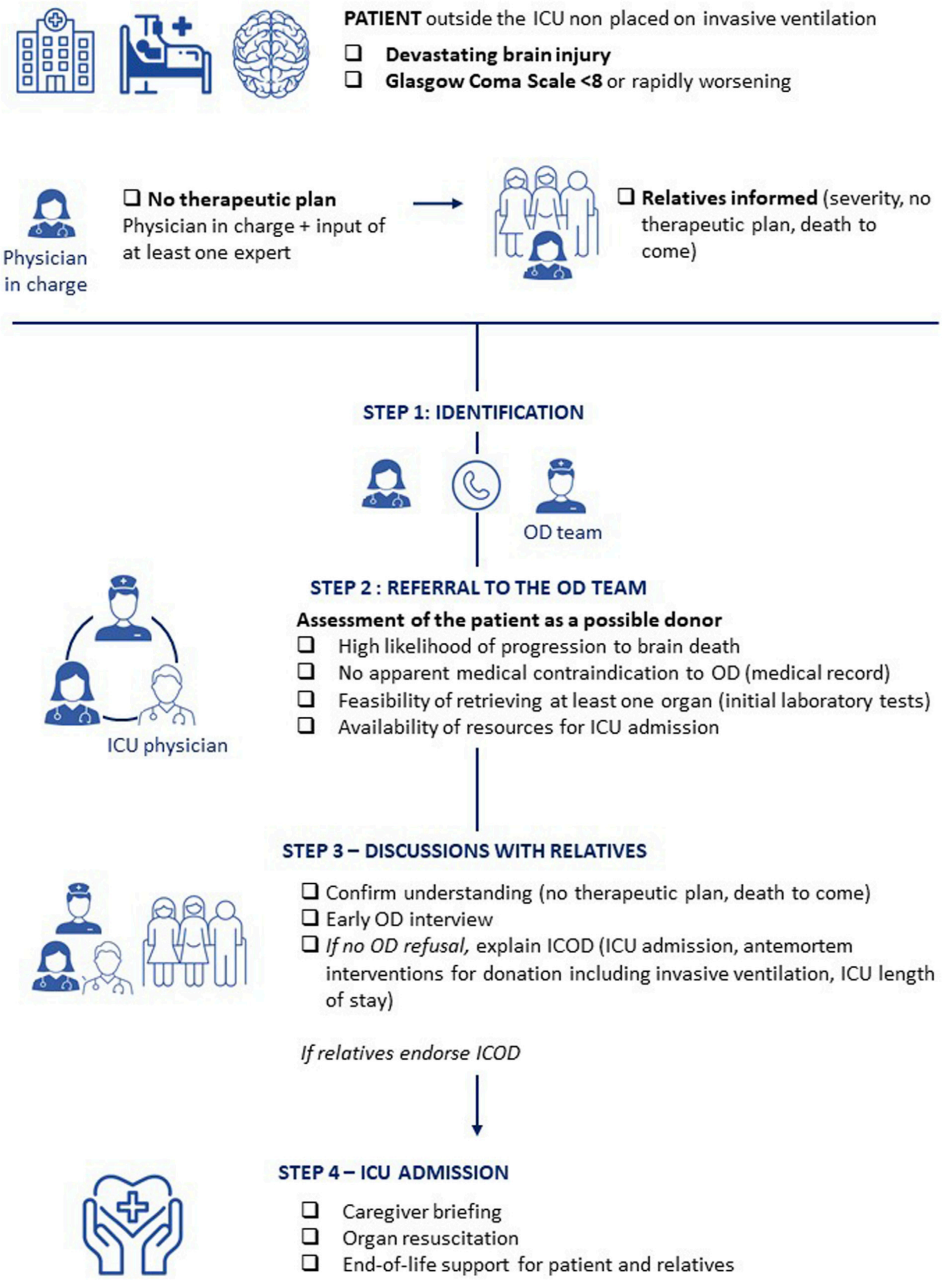
ICOD steps.

The process must follow a series of steps (Figure 1). Two conditions must be met prior to the ICOD process: (1) the absence of therapeutic plan must be established by the physician in charge in the partner department outside the ICU, with input from at least one expert (such as neurologist, neurosurgeon, neurointensivist) and with the involvement of the patient’s caregivers; and (2) the patient’s relatives must have been informed by the senior physician in charge of the diagnosis, the severity and life-threatening nature of the condition, the absence of a therapeutic plan, and the death to come. Caregivers must ensure that the relatives have fully understood this information. From this point, the process proceeds through four steps: (1) identification of the patient as a possible donor by the physician in charge outside the ICU; (2) referral to the OD team, who, in collaboration with the ICU physician, will assess the feasibility of OD; (3) discussions with the relatives, including an early interview regarding OD; (4) admission to the ICU for the sole purpose of OD, while providing appropriate end-of-life care. The physician in charge outside the ICU is encouraged to consult the OD team before making any statement regarding the presence of a contraindication or age limit, in order to avoid inappropriate exclusions that could result in the loss of potential donors.

The *early interview* must be conducted by at least two people, a senior physician–preferably the most experienced physician available for this type of interview - and a member of the OD team. It should take place in a room located close to the patient’s care unit, designed to accommodate all relatives and caregivers, and providing a comfortable, quiet, and dedicated setting for the interview. The timing of the early interview requires caregivers to consider the relatives' experience and the patient’s clinical condition, respecting the steps of the process. This interview must balance transparency in the information provided with acknowledgment of the uncertainties regarding the outcome of the process. It should be carefully prepared by the caregivers, including sharing all available information, defining roles, and agreeing on objectives. A summary of the interview must be documented in the patient’s medical record, and the interview should be evaluated using a debriefing grid. During the early interview, if there is no refusal, the relatives are informed of the need for ICU admission and antemortem interventions for OD, including invasive ventilation, as well as the expected ICU length of stay, which will not exceed a few days. If relatives agree to this process, the patient is admitted to the ICU for the sole purpose of OD while ensuring appropriate end-of-life care.

All caregivers are briefed on the objectives of ICU admission, which include end-of-life care and organ resuscitation in view of potential OD. The presence of relatives should be facilitated throughout this end-of-life phase. Throughout the process, support for the relatives and ongoing reassessment of the ICOD pathway will be provided by ICU and OD team professionals. Relief of suffering must be ensured, even if suffering cannot be assessed due to the patient’s neurological status.

We now propose a more detailed examination of two issues that emerged during the development of these guidelines.

## Are Antemortem Intervention for Donation Ethically Acceptable Within the ICOD Pathway?

Antemortem interventions for donation refer to any clinical procedure or test performed before death with the purpose of facilitating OD, which would not otherwise occur in the absence of consideration of donation One of the main ethical concerns regarding ICOD The first issue is the acceptability of these interventions.

The identification of a patient as a possible donor by the physician in charge outside the ICU is the most critical step in the ICOD process. At this stage, the patient is both a person at the end of his or her life who requires palliative care and a potential organ donor. The initiation of intensive care measures for the purpose of OD, including invasive measures such as invasive ventilation, should be guided by the patient’s wishes. Within the French legal and regulatory framework, the wishes of a patient who is unable to express them must be established by consulting any advance directives, as well as through testimony from the trusted person designated by the patient, or, failing that, from the relatives, regarding any wishes previously expressed by the patient.

In an opt-out system, it is essential to distinguish between presumed consent for OD and presumed consent for antemortem interventions. Presumed consent applies to post-mortem organ procurement and does not necessarily extend to antemortem interventions for donation, such as mechanical ventilation Even in situations where relatives report that the patient explicitly expressed support for OD, whether the patient also agreed to undergo antemortem interventions for donation remains a question that deserves careful consideration, particularly if these interventions may be associated with risks, burden, or suffering for the patient. These antemortem interventions, although intended to benefit recipients, offer no direct benefit to the dying patient and may conflict with the principle of non-maleficence. Some individuals may support OD but nonetheless oppose antemortem interventions for donation. The routine use of such interventions risks undermining public trust if they are perceived as prioritizing recipients’ interests over the dignity of dying individuals. Striking a balance between respecting the patient’s autonomy regarding OD and avoiding harm through invasive antemortem interventions is a delicate and ethically complex challenge.

The most critical situation for questioning the ethical acceptability of these antemortem interventions is when they become necessary for organ preservation before the patient’s wishes can be established through consultation with their relatives.

On the one hand, to support the use of antemortem interventions for donation even before knowing the patient’s wishes, one could invoke the French legal context of an opt-out system. In an opt-out system, as long as it there is no evidence that a patient has expressed opposition to OD, they are presumed to be a donor. Consequently, it could be argued that presumed consent might also extend to antemortem interventions for donation. This opt-out framework allows individuals to easily register their refusal, thereby simplifying and accelerating the OD process while aiming to increase the availability of organs for transplantation. By balancing the need for organ availability with respect for individual autonomy, this system supports the ethical principles of solidarity and medical necessity. This argument is supported by the fact that 80% of people in France say they are in favour of OD after their death [21]. Initiating such interventions in this situation is also a way of considering OD and transplantation as public health priorities. To support these antemortem interventions for donation even before knowing the patient’s wishes, one might argue that admission to intensive care could improve the quality of end-of-life care for patients and their families, or provide additional time to confirm the initial prognostic assessment [12]. However, we challenge these claims, advocating instead for the development of high-quality end-of-life care strategies within emergency or neurology departments, together with the implementation of relevant and robust prognostic tools.

On the other hand, initiating antemortem interventions for donation even before knowing the patient’s wishes carries the risk of violating the patient’s potential refusal of OD or at least their preference not to be admitted to an intensive care unit at the end of their life solely for the purpose of OD, even if they had not explicitly opposed OD itself.

At this stage of the process, the guidelines distinguish between two situations: (1) if the patient does not present with cardiovascular or respiratory failure, the early interview with relatives must take place before any antemortem interventions for donation, such as invasive ventilation, are initiated. These interventions are only undertaken in the absence of any opposition expressed by the patient during their lifetime, in order to comply with the opt-out legal framework, and when the ICOD donation process is also acceptable to the relatives, so as to maintain trust and transparency during this sensitive time; (2) if, however, the patient presents with immediate cardiovascular or respiratory failure, antemortem interventions for donation may be initiated before the patient’s wishes are formally confirmed during the early interview, provided that this decision is made in a transparent and collegial manner involving all caregivers. This approach is more cautious than the Spanish guidelines, which prioritize invasive ventilation and patient stabilization in such circumstances to maximize OD opportunities [2]. The French guidelines leave the possibility for caregivers to consider this strategy as ethically questionable. In any case, the priority must always be the relief of suffering. Invasive mechanical ventilation **is never considered as a means to relieve suffering.** In such cases, these interventions are not performed in the patient’s best interest, particularly when they may prolong the dying process or cause discomfort. Their use may instead be considered within a **separate ethical framework**, namely, the preservation of organ viability for potential OD—which serves the interest of third parties (future recipients).

## How Should cDCD Be Integrated Within the ICOD Pathway?

**FIGURE 2:**
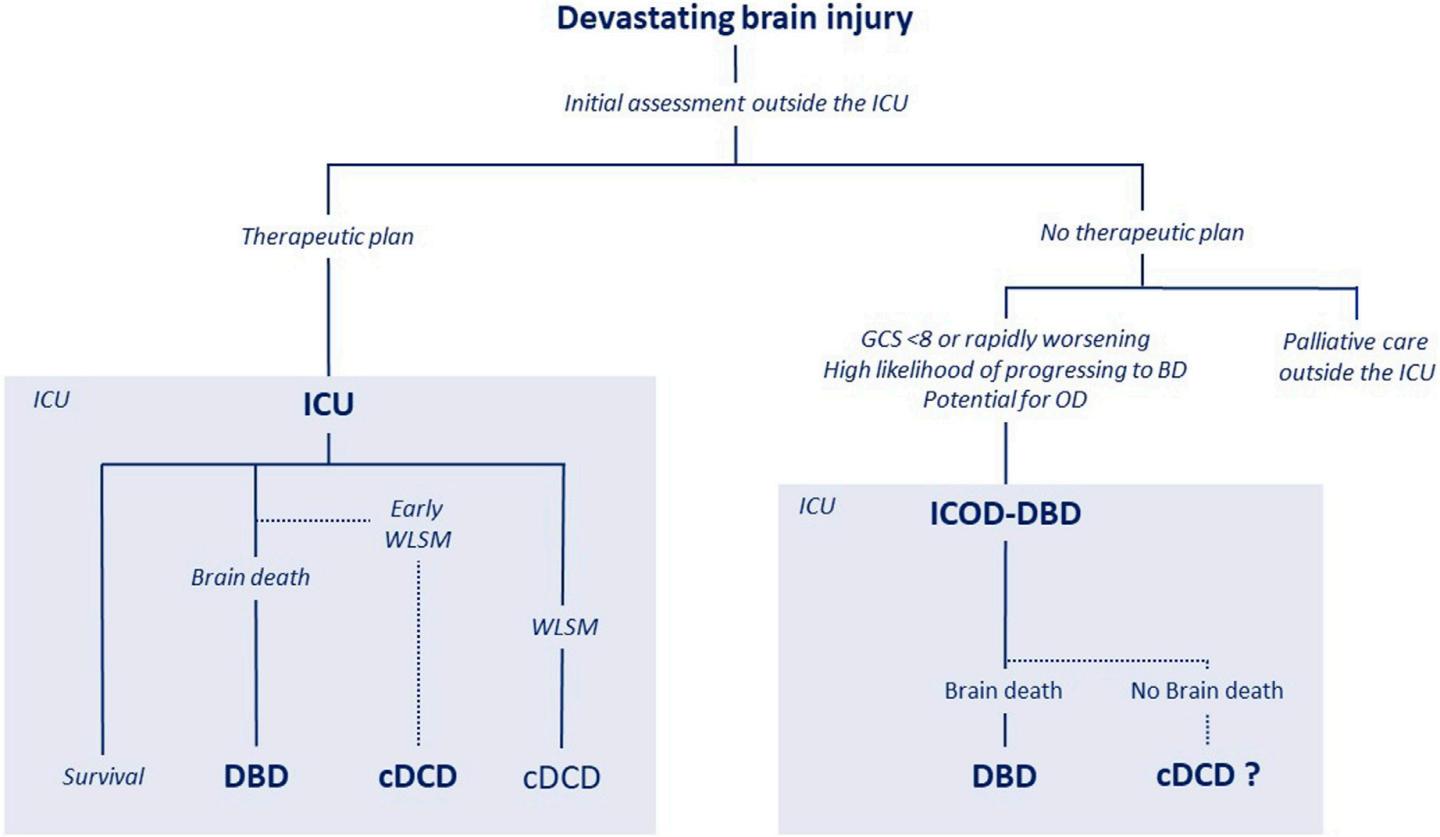
ICOD, DBD and cDCD pathways.

The second issue concerns the ethical acceptability of integrating controlled donation after the circulatory determination of death (cDCD) into the ICOD. For a clearer and more comprehensive understanding of the different pathways discussed throughout this manuscript, readers are invited to refer to Figure 2, which provides a visual summary and clarification of the key processes and decision points described in the text.

Currently, in both Spain and France, the ICOD pathway is primarily considered for patients with devastating brain injury who have a high likelihood of progressing to BD. The aim is to integrate DBD into end-of-life care planning–a process that could be considered as a ICOD-DBD pathway. Only in cases where progression to BD does not occur after several days of observation and reassessment in the ICU may cDCD pathway be considered as a secondary option. This situation may arise as evaluating the probability of progression to BD based on clinical and radiological criteria is not always straightforward, especially in elderly patients. Although advanced age may limit eligibility for certain types of OD, these patients may still be considered for OD under extended criteria or tissue donation. In assessing the likelihood of BD, the intensivist plays a key role. At the end of this period of observation and reassessment, if BD has not occurred, is it ethically acceptable to consider cDCD?

There is currently no international consensus regarding this pathway. The Spanish guidelines explicitly state that if a patient does not progress to BD, cDCD may be considered as an option and be offered to the family [2, 7]. In Spain, cDCD donors account for 20%–30% of actual ICOD donors [7, 22]. In contrast, the French guidelines specify that if the patient does not progress to BD, life-sustaining measures should be withdrawn according to the ICU protocol until death occurs. The French guidelines chose not to explicitly mention the possibility of cDCD as an outcome of an ICOD-DBD pathway, while nevertheless not excluding this alternative.

This choice reflects a precautionary approach intended to limit the risk of a potential shift toward a systematic ICOD-cDCD pathway, which raises two main concerns. First, there is a risk that prognostic evaluation—particularly of neurological outcomes in patients with brain injury—might be influenced by the potential of OD. In other words, OD could shape the prognostic judgment, leading to an early transition from a curative to a non-curative approach before a thorough neurological assessment has been completed. Second, there is a risk of progressively normalizing the integration of OD into end-of-life care, shifting from a palliative care model toward an organ-donation-driven end-of-life framework. Such a development would only be ethically acceptable if it fully respects the patient’s wishes, guarantees the quality of end-of-life care, and preserves the interests of both families and healthcare professionals. This issue is central, as it challenges the future orientation of palliative care more broadly—at least for patients without contraindications to OD. Finally, it seems difficult to envisage integrating cDCD within the ICOD pathway while concerns about transparency and communication regarding cDCD remain significant in France. Should a patient with a high likelihood of progressing to BD be admitted to the ICU and placed on invasive ventilation solely for the purpose of OD? Yes. Should every end-of-life patient be admitted to the ICU and placed on invasive ventilation, only to subsequently withdraw life-sustaining measures under continuous and deep sedation for the sole purpose of OD? Not sure. Including the possibility of ICU admission solely for the purpose of OD within end-of-life care planning remains a challenging issue. Experts believe that the French medical community continues to hold ambivalent views on this issue. In practice, many of those patients will not be eligible for cDCD, given that the current age limit for cDCD in France is 71 years. Most patients under the age of 71 with severe coma related to devastating brain injury are already placed on invasive ventilation during the initial phase of management pending a full assessment of the clinical condition and prognosis. Moreover, the French healthcare system is organized in a specific way, with pre-hospital medical teams and a high capacity of critical care resources.

We have just discussed cDCD as a possible outcome of a DBD-ICOD pathway, in which a patient with a devastating brain injury does not progress to BD for medical reasons after several days in the ICU. However, it is also necessary to consider situations in which a patient with a devastating brain injury could be included directly in a cDCD pathway because a decision to withdraw life-sustaining treatment was made prematurely—whereas continued observation might have allowed progression to DD. This scenario represents an overlap between a DBD-ICOD pathway and, from the outset, a cDCD-ICOD pathway. This second situation raises two concerns: first, the risk of making end-of-life decisions too early, before gathering all prognostic information required for a robust neurological assessment; and second, the risk of contributing to a broader shift from DBD toward cDCD, which, at present, is associated with fewer utilized donors and fewer organs transplanted per utilized donor.

## Conclusion

Organ donation is a public health priority, as the gap between patients awaiting transplants and those identified as potential donors continues to grow [23]. Integrating OD as a routine part of end-of-life care remains challenging. The issues arising from the tension between providing high-quality end-of-life care and the pursuit of OD are complex. Supporting patients and their relatives at the end of life must always take priority. Particular attention should be paid to communication and transparency in order to strengthen public trust in OD.
